# Oral administration of DNA alginate nanovaccine induced immune-protection against *Helicobacter pylori* in Balb/C mice

**DOI:** 10.1186/s12865-024-00602-6

**Published:** 2024-02-03

**Authors:** Arezo Kaveh-Samani, Samaneh Dalali, Fatemeh Kaviani, Tohid Piri-Gharaghie, Abbas Doosti

**Affiliations:** 1 Biotechnology Research Center, Shahrekord Branch, Islamic Azad University, Shahrekord, Iran; 2 Biotechnology Research Center, East Tehran Branch, Islamic Azad University, Tehran, Iran

**Keywords:** *Helicobacter pylori*, Nanovaccine, Alginate, *UreH* gene

## Abstract

**Background:**

Helicobacter pylori (H. Pylori), is an established causative factor for the development of gastric cancer and the induction of persistent stomach infections that may lead to peptic ulcers. In recent decades, several endeavours have been undertaken to develop a vaccine for *H. pylori*, although none have advanced to the clinical phase. The development of a successful *H. pylori* vaccine is hindered by particular challenges, such as the absence of secure mucosal vaccines to enhance local immune responses, the absence of identified antigens that are effective in vaccinations, and the absence of recognized indicators of protection.

**Methods:**

The DNA vaccine was chemically cloned, and the cloning was verified using PCR and restriction enzyme digestion. The efficacy of the vaccination was investigated. The immunogenicity and immune-protective efficacy of the vaccination were assessed in BALB/c mice. This study demonstrated that administering a preventive Alginate/pCI-neo-*UreH* Nanovaccine directly into the stomach effectively triggered a robust immune response to protect against *H. pylori* infection in mice.

**Results:**

The level of immune protection achieved with this nano vaccine was similar to that observed when using the widely accepted formalin-killed *H. pylori* Hel 305 as a positive control. The Alginate/pCI-neo-*UreH* Nanovaccine composition elicited significant mucosal and systemic antigen-specific antibody responses and strong intestinal and systemic Th1 responses. Moreover, the activation of IL-17R signaling is necessary for the defensive Th1 immune responses in the intestines triggered by Alginate/pCI-neo-*UreH*.

**Conclusion:**

Alginate/pCI-neo-*UreH* is a potential Nanovaccine for use in an oral vaccine versus *H. pylori* infection, according to our findings.

**Supplementary Information:**

The online version contains supplementary material available at 10.1186/s12865-024-00602-6.

## Background

*Helicobacter pylori (H. pylori)*, a kind of bacterium with a spiral structure classified as gram-negative, infects almost 50% of the global population. Its main effect is the development of chronic gastritis, which often goes unnoticed. In addition, a small percentage of persons (1–3%) who are infected may develop cancer of the stomach or mucosa-related lymphoid organ lymphoma. Furthermore, a more significant percentage (15–20%) of infected individuals may have disorders associated with systemic inflammation, such as peptic ulcers [1, 2]. *H. pylori* bacteria are transmitted by either the faecal-oral or oral-oral pathways. The bacteria are present in the duodenal and stomach mucosa of unwell individuals. Neutrophils, monocytes, mast cells, dendritic cells (DCs), M1/M2 macrophages, and T and B lymphocytes are attracted to the stomach mucosa as a result of the colonization by *H. pylori* of the mucosa [3, 4]. The presence of *H. pylori* colonization in the stomachs of individuals is associated with a higher abundance of proinflammatory cytokines, including IL-1, IL-6, IL-8, IL-18, TNFα, IFN-γ, and IL-17 A. This correlation is not surprising, as it is to be anticipated [5, 6].

Animal studies have shown that protection against *H. pylori* is mainly achieved via activating Th1 and Th17 immune responses. CD4 + T lymphocytes specific to *H. pylori* and mainly generate IFN-γ and IL-17 A have been detected in the mucous membrane of the stomach and extracellular fluid of individuals who are ill [7, 8]. Nevertheless, although these responses effectively hinder the emergence of apparent diseases in most patients, they lack the strength to halt the colonization process in most individuals. Furthermore, the stomachs of persons infected with *H. pylori* included not only antigen-specific Lymphocytes but also antigen-specific IgA- and IgM-antibody-secreting cells [9]. Research conducted in a highly affected area of Bangladesh found that infants given breast milk with higher levels of *H. pylori*-specific IgA antibodies showed a significant delay in acquiring the infectious illness. This discovery suggests that pre-existing *H. pylori*-specific IgA antibodies may protect against the colonization of the bacterium [10]. Immunizing animals that lack B-cells has shown that IgG antibodies are not essential for protection. Therefore, the role of *H. pylori*-specific IgG in limiting the colonization of bacteria is not well understood [11, 12].

In addition, whereas recipient mice had elevated levels of *H. pylori*-specific IgG, administering serum from vaccinated animals did not protect against the challenge [13]. Evidence indicates that those infected are more prone to have *H. pylori*-specific IgG-secreting cells than those who are not infected. The IgG titers in persons with symptoms and duodenal ulcers did not differ, as reported by other studies [9, 13]. Patients exhibiting symptoms are now treated for *H. pylori* infection by triple therapy. This treatment method entails the prolonged administration of two medicines and a proton pump blocker [14]. Nevertheless, this treatment has several drawbacks, such as poor adherence, adverse reactions, and possibly developing bacterial resistance to the antibiotic. In addition, despite the successful elimination of current infections with triple therapy, re-infections are not avoided. These re-infections often occur in areas where the disease is prevalent and have a median annual occurrence rate of 15–20% [15]. Consequently, a highly effective *H. pylori* vaccination could substantially prevent infection transmission and complement other drugs in a therapeutic context to prevent further infection. Research on the efficacy of vaccines using various antigens, nanoparticles, and delivery techniques has been conducted since the 1990s [1, 16].

In order to efficiently stimulate an immune response at the location of the infection, the most appealing method of vaccination versus *H. pylori* infections would be via the mucosal delivery route, particularly the oral pathway [16, 17]. Nevertheless, the complexity of the immune response triggered by *H. pylori* infection and the lack of secure mucosal nanoparticles have posed challenges in developing vaccines so far [17]. Oral *H. pylori* lysate formulations, whole-cell (WC) killing bacteria, or other combinations of isolated *H. pylori* proteins have been used to investigate candidate vaccines in animal models.

Urease proteins are well-acknowledged as essential antigenic agents in several bacteria. The transit of nickel and maturation of urease are regulated by four proteins: UreE, UreF, UreG, and *UreH* (with *UreH* serving as the equivalent of UreD in other species). The genes were first identified in Klebsiella aerogenes using deletion and complementation approaches. Later, their counterparts were discovered in *H. pylori*, employing a similar approach. The findings of the in vitro pull-down investigation revealed that apo-urease connects with many K. aerogenes urease accessory protein molecules, namely UreD/urease, UreF/UreD/urease, and UreG/UreF/UreD/urease [17, 18]. Yeast-two-hybrid and simultaneous affinity purification methods confirmed the connections between urease and its supporting proteins. The theory proposes that the UreG/UreF/*UreH* combination is responsible for transporting nickel ions into the active site of urease. Moreover, forming the preactivation compound is a pivotal step in the urease maturation. The immunogenic role of urease molecules is well recognized. Therefore, urease subunits have the potential to be appropriate candidates for vaccines [17, 18].

The results consistently demonstrate that significant protection can be achieved by combining a potent mucosal nanoparticle with specific vaccination antigens. However, in the absence of this nanoparticle, none of the tested vaccines have shown any detectable level of protection [1, 17, 18]. The nanoparticles (NPs) enclose the drug inside the particle’s core, providing excellent biocompatibility and protection against drug degradation. Furthermore, much research has been conducted on polymeric nanoparticles (NPs) measuring less than 1000 nm to enhance the oral absorption of medications by facilitating direct interaction with cells in the gastrointestinal tract (GIT). Polymers enhance the attachment of the mucus layer (mucoadhesion) and movement to gastrointestinal (GIT) cells. While polymeric nanoparticles can enclose the medication, protect it from degradation under different circumstances, and enhance its ability to stick to and be absorbed by the gastrointestinal tract, the primary obstacle is precisely delivering the medicine to the colon. Hence, drug delivery methods explicitly targeting the colon have great potential for transporting cytotoxic drugs to the colonic area. The intestinal coating of tablets has historically been used in various methods to inhibit medication release in the upper gastrointestinal tract (GIT). The solubility of enteric polysaccharides is contingent upon the pH level, being insoluble at low pH values and soluble at high pH values in the gastrointestinal tract [17, 18].

Consequently, there has been significant interest in NP-based delivery systems using enteric polymers that induce drug release, specifically in the colon. The delivery systems exhibit stability under acidic conditions, whereas the coating components dissolve under alkaline conditions. As a result, the nanoparticles expand, attach to the colon, and release the active chemicals at the desired locations. Nanoparticles (NPs) can increase the adhesiveness of mucus and prolong the length of contact. Mucoadhesive polymers are used to enhance the adhesiveness of mucus and prolong the interaction with the colon’s lining. Chitosan (CS) and alginate (ALG) are hydrophilic polymers frequently employed to improve mucoadhesive properties using electrostatic attraction, bonding with hydrogen, and hydrophobic interactions with mucin. These polymers have significant promise in this particular application. Thiolation may enhance the mucoadhesive properties of ALG NPs even further [19].

Various mucosal nanoparticles have been used to examine potential proteins that might be associated with *H. pylori*. The combination of cholera toxin (CT) or Escherichia coli heat-labile toxin (LT) with *H. pylori* nanoparticle lysate formulations has been shown to effectively provide resistance against *H. pylori* infections [3, 10, 20]. These potent enterotoxins possess toxicity, rendering them unsuitable for use as mucosal vaccine nanoparticles in humans. Considerable effort has been dedicated to developing mutated forms of these harmless toxins that remain effective when combined with nanoparticles [17–20]. Undoubtedly, the combination of prospective vaccines with alginate nanoparticles has shown promising results in experimental models of *H. pylori* infection [19, 20]. It is essential to investigate nanoparticles that are deemed safe for human consumption. Prior research has shown that alginate amplifies mucosal immune reactions [21]. The current study aimed to evaluate the efficacy of the alginate/pCI-neo-*UreH* DNA vaccination.

## Materials and methods

### Declaration of ethics

The execution of all procedures was conducted in compliance with an animal license and with the approval of the Shahrekord Islamic Azad University Animal Research Ethics Committee. The animals were attended to by the regulations set out by the Department of Health and Shahrekord Islamic Azad University.

### Animals

Female BALB/c mice, aged six to eight weeks, were purchased from Shahrekord Azad University in Iran for all animal studies. The mice were housed in controlled surroundings free of pathogens, known as specialized pathogen-free (SPF) habitats. They were provided with sterile drinking water and food, and the temperature was maintained at a constant 25 °C. The mouse investigations were done using the National Institutes of Health’s Guide for the Clinical and Laboratory Standards Institute Animals (NIH Publications No. 8023). The university’s Ethics Committee approved the Animal Care (Iran). The research was approved by the Ethics Committee of the Islamic Azad University of Shahrekord Branch in Iran (IR.IAU.SHK.REC.1402). Minimal animal suffering was ensured. The inhalation of carbon dioxide (CO_2_) is the primary technique of euthanasia used by the National Institutes of Health (NIH) for small animals, including mice, rats, guinea pigs, and hamsters. The appropriateness of CO_2_ as a euthanasia agent for small animals is based on many essential considerations outlined in the AVMA Guidelines for the Euthanasia of Animals. Compressed carbon dioxide gas is given at a rate that displaces 30–70% of the chamber volume per minute. The animal(s) were put in the chamber and exposed to CO_2_ as directed. The fill rate ranges from about 30–70% of the chamber capacity each minute. According to the American Veterinary Medical Association (AVMA) in 2020, this method will induce quick unconsciousness in animals with little discomfort (AVMA 2020, pp. 31).

### Examining potential vaccine candidates using bioinformatics

Six genome-wide sequences of *H. pylori* strains were discovered as prospective vaccination candidates for this investigation using the Vaxign website (http://www.violinet.org/vaxign/). The 5 participants in the experiment on gene-coding proteins were selected as candidates for immunization.

- An adhesion potential above 0.51.
- Absence of similarity to proteins found in mice or humans.
- A minimum of one transmembrane helix was necessary.

In order to minimize the possibility of an adverse response from the host cell, the potential constituents of the vaccine must not interact with biomolecules found in mice or humans. The proteins were analyzed using Homo sapiens/mice as the host protein and the BLASTp web page on the NCBI website. If the BLASTp online tool confirms the protein specificity, the peptides derived from the *H. pylori* species will be utilized for further investigation. The NCBI used the UNIPORT dataset available at http://www.ncbi.nlm.nih.gov/protein to identify the proteins in the *H. pylori* proteome. The amino acid sequence of these proteins was then stored in FASTA format for future research purposes. The CELLO program, available at http://cello.life.nctu.edu.tw, has been used to determine the specific location of antigens inside the *H. pylori* bacteria accurately. With a Localization Reliability of 1.5, it can identify the location of proteins outside the membrane, whether they are on the outer or inner membrane, and whether the peptide is in the cytoplasm or periplasm. The antigenicity of the selected proteins was assessed using the VaxiJen software, using a threshold of 0.6 (http://www.ddgpharmfac.net/vaxijen/VaxiJen/VaxiJen.html). Bioinformatics analysis was used to examine the physicochemical characteristics of the outer membrane components of *H. pylori*. Subsequently, predictions were made for all possible dominant epitopes of B-cells and T-cells. The B-cell epitopes were forecasted employing the Optimal Antigen Design Tool, a component of bioinformatics software provided by GenScript, a company based in China. Predictions were made for the features of B-cell epitopes, including secondary structure, surface availability, solubility in water, adaptability, and immunological index. The Immune Epitope Database Analysis Tool (IEDB) (https://tools.iedb.org/mhci/) was employed to analyze and forecast the mouse MHC-II genes to identify T-cell epitopes. Proteins possessing more than five B-cell and five T-cell epitopes were evaluated as viable candidates for vaccination.

### Construction of *H. Pylori* DNA vaccines

The *ureH* gene was autonomously generated by the BGI Genomics Company in Shenzhen, China. The *ureH* gene was then incorporated into the plasmids pCI-neo (pDNA) (Invitrogen, USA) to produce pCI-neo-*UreH* (pDNA-*ureH*). This plasmid was verified using DNA sequencing and endonuclease digestion experiments using *Xho*I and *Not*I. The constructs include 20 repetitions of the CpG ODN C274 motif (5’-TCGTCGAACGTTCGAGATGAT-3’) and have a Kozak nucleotide at the N-terminus to enhance the immune response. The Lipofectamine 2000 reagent temporarily introduced the produced plasmids into HEK 293-T cells to validate the eukaryotic expression vector (Invitrogen). The cells were collected and lysed 48 h after transfection. We examined the cellular proteins using a Western blot kit and a rabbit anti-H. pylori polyclonal antibody (pAb) obtained from LifeSpan BioSciences in Seattle, Washington, USA [21, 41].

### Development of Nanovaccines

An alginate nanoparticle synthesis method used a modified water/oil/water dual emulsification evaporation technique. Ultimately, 20 mg of alginate (with a molecular weight of 50,000, obtained from Sigma-Aldrich) was mixed with 2 mL of dichloromethane to create the organic solution. The vaccine candidates were diluted in a solution of PBS and 0.003% tween 80 to create an internal water phase. The dilution included 2 mg of the vaccine candidates. The outer aqueous layer was formed using alginate at a concentration of 2% (w/V). The emulsion was agitated at 1500 revolutions per minute for 24 h at the ambient temperature. The Nanovaccines were produced by freeze-drying the nanoparticles, which had previously undergone three cycles of washing with deionized water and ultracentrifugation (30,000 g for 30 min) to eliminate any excess vaccine candidates. Alginate was effectively used to encapsulate PBS in an amount equivalent to that employed for Nanovaccines, forming alginate-PBS. This substance will serve as a control. The lyophilized nanomaterials were kept at -80 °C before use [17–21].

### Scanning electrode microscope (*SEM*), Zeta Potential, and particle size

A particle size analyzer determined the particle size, polydispersity index (PDI), and surface charge. The nano vaccines were suspended in filtered, purified water and introduced into a cuvette with continuous agitation to evaluate their size and surface charge. The median value of triple tests was used to ascertain each sample’s dimension (expressed in nanometers) and charge density (expressed in millivolts). The morphology of Nanovacines was analyzed using high-resolution scanning electron microscopy (SEM) using a Hitachi HT7700 Exalens instrument. The chemical was deposited onto a metal plate, specifically a carbon-coated copper grid with a mesh size 200. The grids were coated with phosphotungstic acid and then air-dried for 10 min before the SEM analysis [21, 41].

### Electrophoretic mobility

The electrophoretic mobility of the Alginate/pCI-neo and Alginate/pCI-neo-UreH nanoparticles was assessed using gel electrophoresis. The resultant solutions, 4 ml of loading buffer, and ethidium bromide were loaded onto a 1% agarose gel (1% w/v) with ethidium bromide.

### The efficiency of encapsulation and vaccine loading

Each sample was supplemented with complexes comprising 4 ng of Alginate/pCI-neo-UreH and Alginate/pCI-neo. Initially, the alginate polymer was rinsed with dichloromethane to remove the enclosed DNA. Subsequently, the samples were placed in a 1.5 ml cuvette and exposed to 25ul of 1X OliGreen (OG) fluorescent nucleic acid stain for 5 min at room temperature in a lightless environment. After the incubation period, Millipore water was added to the cuvette to bring the total volume up to 500ul. The fluorescence intensity was determined by measuring the emitted light at 520 nm after stimulating the sample with light at a wavelength of 490 nm using a luminescence spectrophotometer [21, 41].

### Analyzing the stability and release of nanovaccines

The liberation of the vaccine from the alginate was examined by dialysis. The dialysis tubing was immersed in distilled water for 24 h. The dialysis bag involved 0.5 ml [10 mg] of Nanovaccine and a 0.5 ml PBS reference solution. Conical flasks, each holding 75 ml of distilled water and dialysis bags, were agitated at 50 revolutions per minute in distilled water at a temperature of 37 °C. A volume of 5 ml was periodically taken from the receptor media at intervals of 1, 2, 4, 6, 12, and 24 h. The concentration of Nanovaccine was then determined using spectrophotometry at a wavelength of 281 nm. At 37 °C, the specimens were partitioned into smaller segments and the original medium was substituted with the new medium. The dispersion of the compounds was then established by using several mathematical models that describe the diffusion rate. This method was used to monitor the consistency of the spread of several Nanovaccine compositions during intervals of 7, 14, 21, 28, 35, 42, 49, and 56 days over a two-month storage period at 25 °C [21, 41]. Two treatment groups, one with nuclease and one without nuclease, were studied for 72 h.

### Cytotoxicity research

The CCK-8 experiment was used to quantify the cytotoxic impact of Nanovaccines on the HEK-293 cell line. The HEK-293 cell lines were seeded at a density of 1 × 10^5^ cells per well in 6-well plates using 100µL of the specified medium for the cell viability assay. Subsequently, cell adhesion was enhanced by an extended incubation period at 37 °C in a humid atmosphere containing 5% CO_2_. The cell lines were subjected to different concentrations of Nanovaccine (ranging from 1.25 to 80 µg/mL) for 24 h. The medium was combined with 10µL of CCK-8 solvent and incubated for two hours. The optical density (OD) values were determined at a wavelength of 450 nm using a spectrophotometric technique (Bio-Rad iMark) [21, 41].

### An in-vitro examination of nanovaccines in *E. Coli* BL21 (DE3) employing reverse transcriptase-PCR and Western blot analysis

Reverse transcriptase-PCR was used to measure the relative mRNA expression levels of the *ureH* DNA vaccines in the *E. coli* BL21 (DE3) strain (RT-PCR). Total RNA was extracted after 36 h using an RNA isolation TRIzol kit (Sisco Research Laboratories [SRL], India). According to the YTA Kit Protocol, cDNA was created (Yekta Tajhiz, Iran). The GAPDH gene (Table 1) was utilized as an internal control throughout the RT-PCR procedure. Finally, agarose gel electrophoresis was used to evaluate the PCR result.

**Table 1:** List of primers used in this study

Gene	Primer sequence	Accession Number
***IFN- γ***	F: 5’- GCCTAGCTCTGAGACAATGAACG − 3’ R: 5’- GCCAGTTCCTCCAGATATCCAAG − 3’	NM_008337.4
***IL17***	F: 5’- CTACAGTGAAGGCAGCAGCGATC − 3’ R: 5’- CTTTCCCTCCGCATTGACACAG − 3’	NM_010552.3
***GAPDH***	F: 5ˊ-ACCTTGGAAATAAATGGGAAG-3ˊ R:5ˊ- CTTCTGTGTTGCTGTAGTTGC-3ˊ	NM_001289726.1
***UreH***	F: 5’-ATGAACAGTAAATTATCC-3’ R: 5’-GAATTTGCTCTGCACGACA-3’	CP018823.1

Rojan Azma Company in Iran investigated antigen purification utilizing Western blotting and antigen sandwich approaches to evaluate antigen production. The Recombinant Nanovaccine samples and control group samples were collected in RIPA buffer, subjected to electrophoresis on 12% SDS PAGE gels, and then transferred onto nitrocellulose (GE Amersham Biosciences, Piscataway, NJ, USA). The proteins were detected using western blotting, which included employing primary antibodies (specifically monoclonal Abs against r-*ureH*) at a concentration of 1/1000. The blotting process was carried out overnight at a temperature of 4 °C. The primary antibodies were obtained from Santa Cruz Biotechnology in Santa Cruz, CA, USA. The antibodies were immobilized onto nitrocellulose sheets and tagged with HRP. The first antibodies [21, 41] were labelled and identified using Anti-Abs, HRP (Santa Cruz Biotechnology), and ECL reagents.

### Grouping of mice

For this study, 120 female Balb/C mice, ranging in age from 6 to 8 weeks and weighing between 18 and 20 g, were used. Animals were administered a vaccination group by oral immunization, with 100 µg of pDNA. 100 mice were divided into 5 groups of 20, with an extra 20 as negative controls (*n* = 20) and receiving PBS. The number of mice utilized by each group is provided in Table 2. Each mixture was administered to a batch of 20 mice using an oral pipette, with each administration repeated 3 times. The medication was administered in three cycles, each lasting three consecutive days (days 0–2, 14–16, and 28–30), with a 15-day interval between each cycle. The animals in the positive control groups were administered formalin-killed *H. pylori* Hel 305 vaccines.

**Table 2:** The number of mice used in this experiment

Group number	Injection composition	Number of mice	Average weight of mice (gr)	Type of injection	immunization day
1	Alginate/pCI-neo-*UreH*	20	19.7 ± 0.3	*oral*	0–2, 14–16, 28–30
2	Alginate/pCI-neo	20	19.5 ± 0.7	*oral*	0–2, 14–16, 28–30
3	Alginate	20	19.6 ± 0.6	*oral*	0–2, 14–16, 28–30
4	pCI-neo-*UreH*	20	19.3 ± 0.3	*oral*	0–2, 14–16, 28–30
5	formalin-killed *H. pylori* Hel 305	20	19.6 ± 0.4	*oral*	0–2, 14–16, 28–30
6	PBS	20	19.7 ± 0.8	*oral*	0–2, 14–16, 28–30

### Immunization, *H. Pylori* challenge, and bacterial quantification in the stomach

The mice groups received three vaccines on days 0–2, 14–16, and 28–30. The mice were divided into two groups for immunization. The first group received PBS alone as a negative control. In contrast, the second group received 200 µl of sterilized Phosphate-buffered saline (PBS) containing 1 × 10^9^ formalin-killed *H. pylori* Hel 305 as a positive control. Vaccinations were administered to various groups according to the guidelines outlined in Table 2. On the 28th day, an analysis was conducted to evaluate cellular and antibody responses. The animals were vaccinated thrice on days 0, 15, and 30 in preparation for the challenge experiments. The mice were administered a challenge of 3 × 108 live *H. pylori* SS1 using a feeding syringe under anaesthesia two weeks after their last vaccination. As stated in the earlier study conducted by Raghavan et al., the animals were euthanized three weeks following the challenge, and the number of bacteria in the stomach was measured by quantitative culture. The quantitative culture method was used to determine the contents of the stomachs [22].

### Analysis of cellular immune responses and splenocyte proliferation

As stated before, mice were euthanized, and their cellular responses were examined. MLN and splenocytes were cultivated at a density of 1 × 10^6^ cells per milliliter. The cells were stimulated with boiled Hel 305 lysates antigens (1 µg/ml) or pure MP305 (1 µg/ml) for 72 h at 37 °C with 5% CO_2_. The Mice IFN-γ and IL-17 DuoSet Immunoassay kits were used to measure the levels of IFN-γ and IL-17 in the collected supernatants 72 h after stimulation. The cells were stimulated using 1 µCi of [3 H] thymidine for 7 h to induce splenocyte growth (Amersham Bioscience Buckinghamshire, UK). A liquid scintillation counter measured the quantity of 3 H incorporation in cellular DNA. The DNA was harvested using a cell harvesting method (Skatron) using glass fibre filters (Wallac) from Beckman, Sweden.

### Collection of faecal and serum samples

Five recently excreted faecal pellets were collected from five mice and kept on ice for four hours in 500 µl of a cold solution made up of 0.1 mg/ml Soybean trypsin inhibitor, 1% Bovine Serum Albumin, 25 mM ethylene diamine tetra acetic acid (EDTA), 1 mM PEFABloc, and 50% Glycerol in 1 PBS. Following the weighing and emulsification process, the samples were centrifuged at a speed of 15,400 times the force of gravity for 5 min at a temperature of 4 degrees Celsius. The blood of the mice was obtained by puncturing their tail veins. The samples were subjected to centrifugation with a force of 9200 times the acceleration due to gravity for 10 min after an overnight coagulation period at a temperature of 4 °C. The serum was stored at a temperature of -20 °C.

### Preparation of intestinal tissues

The Perfusion-Extraction (PERFEXT) technique collected IgA responses from the stomach, jejunum, ileum, and colon. The mice were euthanized, and then a solution of 0.1% Heparin-sulphate (Sigma Aldrich) in PBS was injected into their hearts and caudal mesenteric arteries, with a volume of 20 ml. The intestines were washed in PBS and placed in 270 µl of ice-cold sample buffer containing 0.1 mg/ml STI, 0.05 M EDTA, 1mM PEFABloc, 0.1% BSA, and 0.05% Tween 20 in 1 PBS. 30 µl of 20% quillaja bark saponin from Sigma-Aldrich was added to each tube and incubated at 4 °C overnight. The liquid portion was obtained by spinning the mixture with a force of 14,000 times the acceleration due to gravity for 10 min at a temperature of 4 °C. The resulting supernatants were stored at -20 °C [21, 41].

### Measurement of antigen-specific antibody responses

The MP305-specific ELISA assay was used to quantify the levels of antibody titers. Greiner BioOne’s high-binding 96-well plates were coated with 50 µl of pure MP305 in PBS at a concentration of 5 µg/ml for overnight incubation. The ELISA plates were coated with a solution of 0.1% BSA in PBS and incubated at 37 °C for 30 min. Before coating, the plates were washed thrice with a wash buffer consisting of PBS and 0.05% Tween 20. The plates were washed twice with PBS to remove the blocking solution. After rinsing, various substances (such as excrement, tissues, or blood serum) were added and progressively diluted throughout the plate using a solution containing 0.1% BSA and PBS-T. The plate was then left to incubate at room temperature for 90 min. Previously, PBS was used to clean the plates. The HRP-conjugated anti-mouse IgA (1:1500) or HRP-conjugated anti-mouse IgG (1:4000) from Southern Biotech were added to each well and incubated at 4 °C overnight [21, 41]. The plates underwent three rounds of cleaning in a wash buffer before a final cleaning in PBS. A solution of phosphate citrate with a concentration of 0.1 M and a pH of 5 was prepared. This solution included 4 µl of H_2_O_2_ per 10 ml of o-phenylenediamine dihydrochloride substrate from Sigma Aldrich. Then, 50 µl of this solution was added to each well. The plates were incubated for 24 h prior to terminating the reaction with the addition of 25 µl of 1 M H_2_SO_4_. Subsequently, the Microplate Reader was used to collect data on the absorption at 492 nm. Antibody concentrations were expressed as endpoint titers, calculated by determining the highest dilution of serum or faecal pellet supernatants that produced a reading over the threshold value plus two standard deviations. The threshold was determined based on the average OD492 measurements obtained from the control samples [21, 41].

### Statistics

The statistics shown in each graphic are mathematical averages. The data were standardized for antibody investigations using control groups treated with PBS. A one-way analysis of variance detected significant differences between treatments, and the significance level was evaluated using the Dunetts and Tukey multiple comparison test. A Mann-Whitney test was used to evaluate the statistical significance of two specific treatments. Prism5, developed by GraphPad, was the primary tool for all statistical studies. P values below 0.05 were considered statistically significant.

## Results

### A possible vaccination candidate has been identified

The Vaxign dataset had an overall number of twenty proteins that displayed remarkable characteristics. Afterwards, 14 proteins with the potential to cause illness were chosen. Following an analysis of protein antigenicity, the pool of prospective vaccine candidates was narrowed to 5. The Uniport dataset verified the existence of five proteins in *H. pylori*, and their respective accession numbers on the NCBI website were chosen. The proteins were classified into five categories according to the results obtained from the CELLO online application: extracellular, outer membrane, periplasmic, inner membrane, and intracellular proteins. In order to reduce the chances of the vaccine interacting with host cells, proteins for *H. pylori* were chosen depending on their particularity, which was verified by BLASTp protein analysis. Table 3 displays the first evaluation findings for 5 *H. pylori* proteins. The UreH protein was chosen for immunogenicity based on its exceptional solubility, adaptability, and antigenic index of 0.70, as indicated by the preliminary screening results.

**Table 3:** Preliminary screening and identification of protein

Protein	Symbol	Accession numbers	CELLO analysis		Vaxign			VaxiJen
			LOCALIZATION	Score	Adhesin Probability	Similarity to Host Proteins	Y/N	
Urease H	*ureH*	AAA25026.1	Cytoplasmic	4.161*	0.279	Human	N	0.56
						Mouse	N	
						Pig	N	
flagellar assembly protein H	fla H	ADU84372.1	Cytoplasmic	3.492 *	0.854	Human	N	0.48
						Mouse	N	
						Pig	N	
YqgF family	YqgF	EMH24019.1	Cytoplasmic	3.565 *	0.688	Human	N	0.39
						Mouse	N	
						Pig	N	
outer membrane beta-barrel protein HofF	HofF	TLR92415.1	OuterMembrane	4.796 *	0.492	Human	N	0.54
						Mouse	N	
						Pig	N	
Cytotoxin-associated gene A	CagA	QGM12958.1	OuterMembrane	3.462 *	0.291	Human	N	0.46
						Mouse	N	
						Pig	N	

* More than 1.5

### Generating and identifying recombinant pCI-neo-*UreH*

**Fig. 1:**
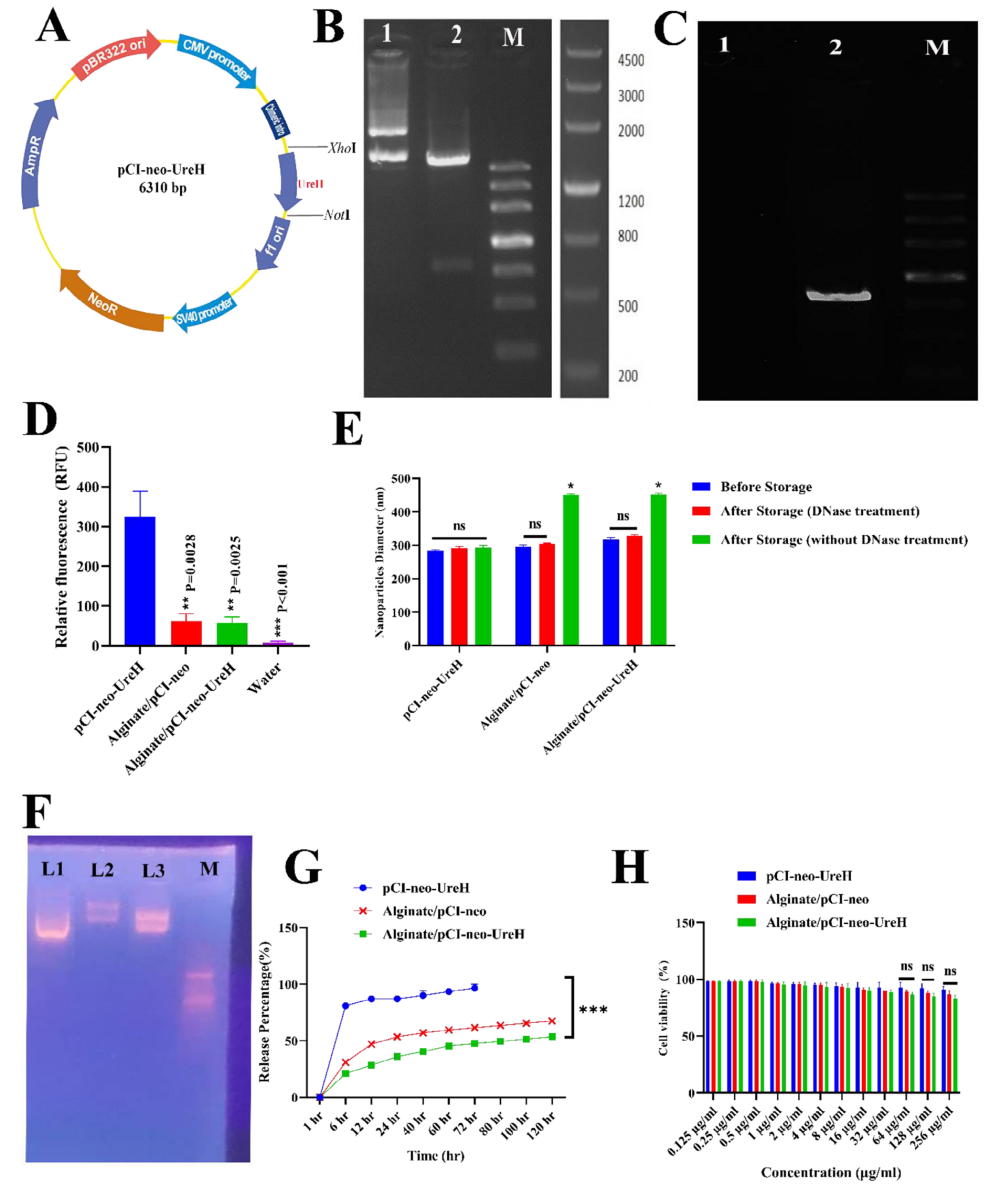
**(A)** The *UreH* gene was inserted into the pCI-neo expression vector using *Xho*I and NotI. **(B)** The effective synthesis of the recombinant plasmid was verified using electrophoretic extraction of the *UreH* digestion fragments. 1: pCI-neo-*UreH* recombinant plasmid before digestion, 2: pCI-neo-*UreH* recombinant plasmid after digestion, M: Marker III DNA Ladder. **(C)** Co-transfection of the recombinant constructs to the HEK 293-T cells resulted in 810 bp bands after PCR analyses using specific primers. 1: Negative control, 2: 810 bp bands of *UreH* gene, M: Marker III DNA Ladder. **(D)** The number of NPs in each interaction determines the relative fluorescence of pDNA in each sample. Free-pCI-neo has the most fluorescence. Fluorescence intensities decrease in the Alginate/pCI-neo-*UreH*. **(E)** Nanoparticle size measurement following storage by freezing at − 20 °C for 1 month. **(F)** Electrophoretic movement of different formulations of nanovaccines. Lane M: negative control; Lane 1: Free-pCI-neo; Lane 2: Alginate/pCI-neo-*UreH*; Lane 3: Alginate/pCI-neo. **(G)** In vitro release kinetic of different formulations of nano vaccines at pH 7. **(H)** Cytotoxicity of several nano vaccine formulations on HEK-293 cells after 24 h (*n* = 3; mean ± standard error; ns: not significant; *p ≤ 0.05; **p ≤ 0.01, ***p ≤ 0.001)

The *UreH* gene was inserted into the pCI-neo expression vector to generate the DNA vaccine construct depicted in Fig. 1A. The recombinant plasmid’s genetic composition was used to evaluate the efficacy of the cloning process. Moreover, DNA testing revealed that the virulence gene sequence of the recombinant plasmid was 100% similar to that of the *H. pylori* bacteria. *Xho*I and NotI were used to digest the generated plasmid. The effective synthesis of the recombinant plasmid was verified using electrophoretic extraction of the *UreH* digestion fragments at 810 bp (Fig. 1B). Blast was used to check the outcomes of the sequencing of recombinant plasmids. Recombinant vectors with an E-value of 8e-41 and 99.21% Per Ident were similar to the target bacteria.

In this study, HEK 293-T cells were transfected with the vector containing GFP and GFP-free vector for producing control cells. Twenty-four hours after lipofection, cells were inspected using fluorescence microscopy, and their photo was captured. Cells containing a GFP vector had green dots, while cells with a GFP-free vector did not have green dots. Green dots show the cells carrying the vector GFP, indicating the lipofection’s success. Co-transfection of the recombinant constructs to the HEK 293-T cells resulted in 810 bp bands after PCR analyses using specific primers (Fig. 1C).

### Physical-Structural Assessment of Nano vaccines’

The characteristics of several classes of Nano vaccines are summarized in Table 3. Transmission electron microscopy (TEM) techniques were used to assess the shape and dimensions of the Nano vaccines. Alginate/pCI-neo-*UreH*, Alginate/pCI-neo, and Alginate nanoparticles (NPs) all appeared homogenous with consistently double spherical morphologies, according to the TEM research. Alginate/pCI-neo-*UreH*, Alginate/pCI-neo, and Alginate NPs had diameters of 384.24 ± 10.17 nm, 383.19 ± 9.46 nm, and 292.93 ± 21.49 nm, respectively. These nano-range dimensions (100–400 nm) and acceptable uniformity were determined by the particle size analysis (PDI range of approximately 0.187 to 0.328). The zeta potentials of the Alginate/pCI-neo-*UreH*, Alginate/pCI-neo, and Alginate NPs were 3.81 ± 1.45, 3.68 ± 1.53, and 3.21 ± 2.74 mV, respectively, as shown in Table 4. During the saline solution stability study for the Alginate/pCI-neo-*UreH* Nano vaccines, no aggregation of the nanoparticles was found (Table 5).

**Table 4:** Morphological features of nanovaccines compared to blank group

Formulations	Polydispersity index	Zeta Potential (m.v)	Vesicle size (nm)	EE (%)
Alginate	0.187 ± 0.04	3.21 ± 2.74	292.93 ± 21.49	---
Alginate/pCI-neo	0.281 ± 0. 07	3.68 ± 1.53	383.19 ± 9.46	72.34 ± 2.14
Alginate/pCI-neo-*UreH*	0.328 ± 0.05	3.81 ± 1.45	384.24 ± 10.17	79.54 ± 1.21

average ± SD was use to analysis

**Table 5:** Storage stability of the Alginate/pCI-neo-*UreH* NPs in saline solution at 4 °C

Time (h)	No treatment with nuclease		Nuclease treatment	
	Particle Size (nm)	PDI	Particle Size (nm)	PDI
0	384.24 ± 10.17	0.328 ± 0.05	384.24 ± 10.17	0.328 ± 0.05
24	395.10 ± 7.14	0.367 ± 0.04	387.91 ± 8.34	0.335 ± 0.43
48	404.31 ± 1.34	0.404 ± 0.007	389.42 ± 3.42	0.339 ± 0.37
72	421.14 ± 2.05	0.415 ± 0.024	389.39 ± 6.34	0.339 ± 0.86

**Notes**: At 4 °C, the Alginate/pCI-neo-*UreH* NPs’ stability in saline solution was assessed. A particle size analyzer measured the particle size and PDI daily for 72 h (Anton Paar, Graz, Austria). The results demonstrated the absence of nanoparticle agglomeration

### Nanovaccines’ Encapsulation Effectiveness and Release studies

The experiment demonstrates the degree of pDNA encapsulation by the fluorescence intensity of the OliGreen nucleic acid indicator. The fluorescence can only reach its highest level if a free vector is present in the sample. The absence of fluorescence suggests that the pDNA has reached a level of complexity that prevents its interaction with DNA dye and subsequent fluorescence. A p-value of 0.0001 suggests a significant decrease of 70.43% in the maximum intensity for Alginate/pCI-neo-*UreH*. Once the plasmid DNA (pDNA) has undergone digestion, the optimal ratio for preserving and transmitting the whole DNA load is achieved (Fig. 1D).

The size of the nanoparticles was subsequently determined after a month-long period of freezing at a temperature of -20 °C. The growth in nanoparticle size during storage (Fig. 1E) may have been caused by the gravitational attraction between negatively and positively charged particles, namely negative charges free-pCI-neo or pCI-neo-*UreH* at the Alginate exterior.

Figure 1F illustrates the presence of Plasmid bands in pairs of electrophoresis lanes 2, demonstrating the encapsulation of pCI-neo-*UreH* inside Alginate nanoparticles. In addition, lane 3 illustrates the encapsulation of free-pCI-neo-*UreH* inside Alginate nanoparticles. Due to its greater size, alginate-encapsulated pCI-neo-*UreH* exhibits a slower movement (lane 2) and a more prominent band than free pCI-neo-*UreH*. Lane 1 exhibits a faster movement and shows the presence of free-pCI-neo without the *UreH* gene. The work included the creation of highly cationic nanoparticles (Table 4).

Based on the investigation of release kinetics of Alginate/pCI-neo-*UreH*, it was observed that Alginate-based pCI-neo-*UreH* exhibited more excellent stability in a pH 7.0 environment (Fig. 1G). This characteristic might potentially avoid premature termination of Alginate/pCI-neo-*UreH* and ensure the presence of pDNA in a typical biological setting. Furthermore, the inclination towards low pH facilitates the liberation of Alginate/pCI-neo-*UreH* from acidic endosomes after being taken up by cells. Figure 1G illustrates a two-phase release strategy, where 25% and 29% of Alginate/pCI-neo-*UreH* and Alginate/pCI-neo-NPs are released in the first seven-hour phase. The pDNAs released at the early stage are undoubtedly not encapsulated. During the first stage, 75% of the Alginate NPs were released, followed by the complete release of all Alginate NPs over 72 h. The Alginate/pCI-neo-*UreH* and Alginate/pCI-neo formulations exhibited release rates of 65% and 69% after 120 h, respectively, indicating a progressive release pattern for both methods.

### Nanovaccines’ cytotoxic effect on HEK-293 cells

HEK-293 cells were used to assess the toxicity of several nanovaccine formulations. Toxicity research was done on HEK-293 cells, where several Nanovaccine formulations were tested at concentrations ranging from 0.125 µg/mL to 256 µg/mL. The investigation was repeated 24 times to evaluate the effects of time and dosage on toxicity. The vitality of HEK-293 cells remained unaltered by any concentration of Nanovaccines during the whole research period, as shown in Fig. 1H. This indicates that the Nanovaccines were non-toxic to HEK-293 cells, confirming their safety and compatibility for further examination in living organisms. Figure S1 (Supplementary material) illustrates the population growth of cells subjected to various formulations.

### Expression of recombinant plasmids in mRNA and protein level

**Fig. 2:**
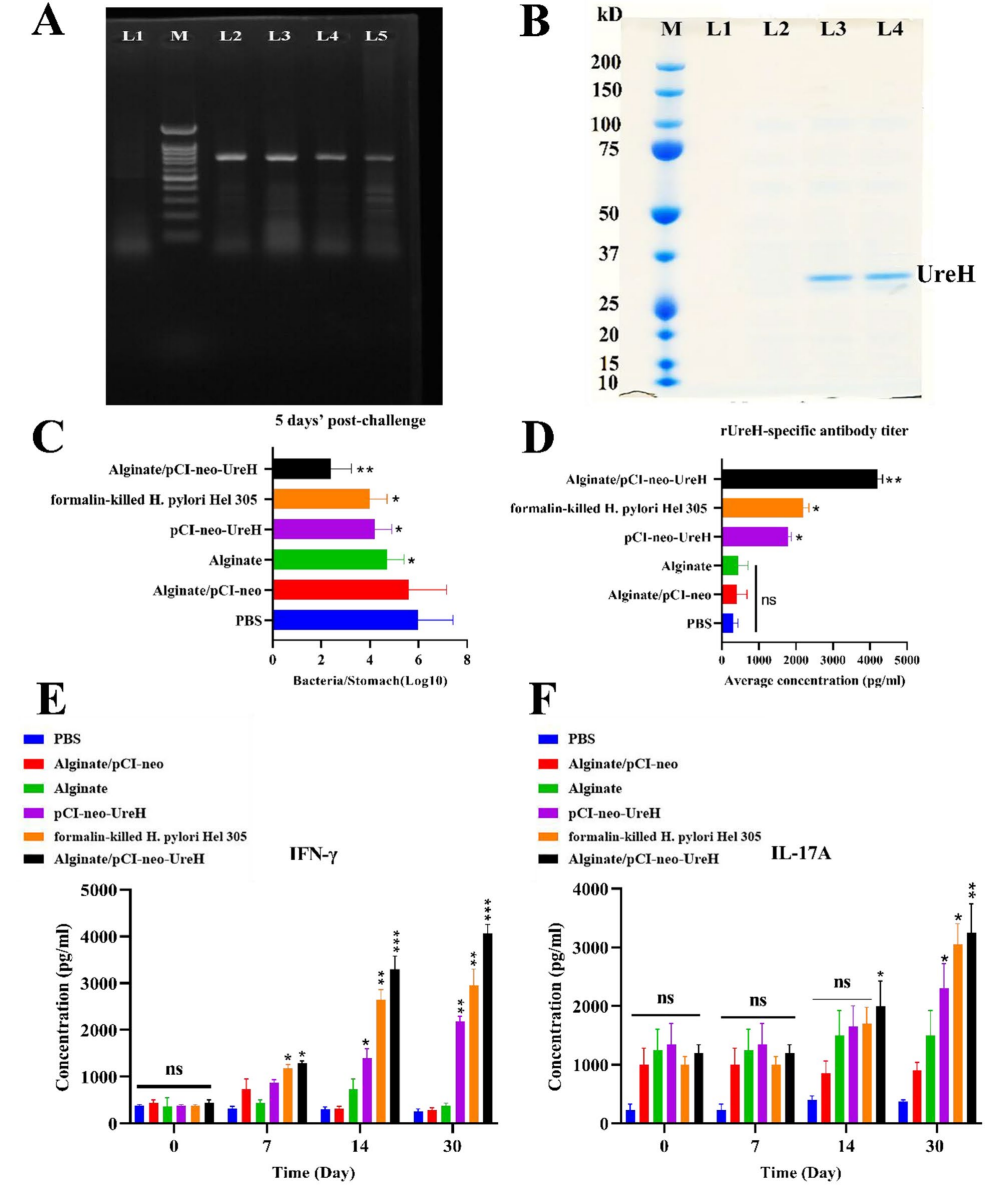
**(A)** Transcription of the recombinant vaccine at mRNA level. L1: Negative control; L2 and L3: Alginate/pCI-neo-*UreH*; L4 and L5: pCI-neo-*UreH*; M: 100 bp DNA marker. **(B)** Expression of recombinant Alginate/pCI-neo-*UreH* in protein level. L1: Negative control; L2: Alginate/pCI-neo; L3: Alginate/pCI-neo-*UreH*; L4: pCI-neo-*UreH*; M: protein marker. **(C)** Bacterial burdens in the stomach tissues of BALB/c mice 5 days’ post-challenge. The stars (*) are according to the PBS group. IgG (**D**), IFN-γ (**E**) and IL-17 A(**F**) levels were found in the splenocyte supernatants of the control and immunized groups. PBS was used as a control. * *p* < 0.05, ** *p* < 0.01, *** *p* < 0.001

The DNA vaccines’ relative amounts of mRNA expression were determined using reverse transcriptase-PCR (RT-PCR). In contrast to the other groups, the Alginate/pCI-neo-*UreH* group had sharper bands, suggesting that Alginate NPs impacted plasmid distribution to host cells. Some groups with weaker bands suggest that DNA vaccines’ capacity to reach host cells is limited. Relative expression levels in Alginate/pCI-neo-*UreH* were substantially higher than pCI-neo-*UreH*. On the other hand, DNA vaccines overcame this limitation by encasing pDNA in Alginate (Fig. 2A). The Western blot analysis by Rojan Azma also revealed that the targeted antigens were expressed at the protein level. Protein-level antigen expression also revealed that Alginate/pCI-neo-*UreH* enhanced protein concentration compared to pCI-neo-*UreH* (Fig. 2B).

### *H. Pylori* infection is prevented by oral immunization with the Alginate/pCI-neo-*UreH* vaccine

Developing an efficient vaccination depends on identifying a safe and orally active vaccine that induces protective mucosal and systemic *H. pylori*-specific immune function. Mice were immunized with several vaccines to assess the efficacy of the oral Alginate/pDNA vaccine. Mice were exposed to live *H.pylori* two weeks following the second of two immunization cycles. By quantifying the microbial count in the stomach five days after the challenge and comparing it to the bacterial load in unvaccinated mice, researchers could evaluate the impact of vaccination on both acute and chronic illness (infection control group). Alginate/pCI-neo-*UreH* oral immunization significantly reduced bacterial burdens in stomach tissues compared to the infection control group (Fig. 2C) at levels equivalent to those found in mice immunized with pCI-neo-*UreH* and other groups. Mice inoculated with the Alginate/pCI-neo-*UreH* vaccine showed a 20-fold reduction compared to the infection control groups five days after the challenge. Furthermore, our study demonstrated that Free-Alginate and Alginate/pCI-neo were necessary to provide noticeable immune protection. Consequently, vaccination with these vaccines did not offer any resistance against the challenge. Bacterial loads in the blood of mice at 5 days’ post-challenge was shown in supplementary materials (Figure S2). Together with these local responses, orally, animals given the Alginate/pCI-neo-*UreH* formulation exhibited higher serum antigen-specific IgG antibody titers (Fig. 2D) and increased splenocyte proliferation following stimulation compared to the infection control group 3 weeks after exposure. The presence of *H. pylori*-specific IgG antibodies does not provide immunity during vaccination. Instead, a high IgG titer indicates that the vaccine was sufficient. Interestingly, compared to the infection control group, mice given the Alginate/pCI-neo-*UreH* vaccine significantly increased IFN-λ gene expression in their stomach tissue (Fig. 2E). Also, three weeks after infection, there had been no such significant increase in IL-17 A gene expression (Fig. 2F).

### Alginate/pCI-neo-*UreH* oral immunization promotes a systemic and mucosal Th1 reaction

T-cell responses, particularly Th1 and Th17 cells, were essential for vaccine-induced immunity versus infection with *H. pylori*. The observed increase in IFN-γ genetic expression in stomach organs after challenge with *H. pylori* and immunization with Alginate/pCI-neo-*UreH* leads to the activation of a local Th1 reaction. Mice were Orally immunized twice with Alginate/pCI-neo-*UreH* and other vaccination groups to measure vaccine-induced antigen-specific Th1 and Th17 reactions. Cellular responses in spleens and mesenteric lymph nodes (MLN) were examined. *H. pylori* strain 305 membrane protein (MP305) activated isolated splenocytes and MLN cells in vitro two weeks after the previous vaccination. In contrast to splenocytes from mice immunized with the other groups, Alginate/pCI-neo-*UreH*-vaccinated animals showed significantly greater levels of IFN-γ in their supernatants from restimulated splenocytes.

**Fig. 3:**
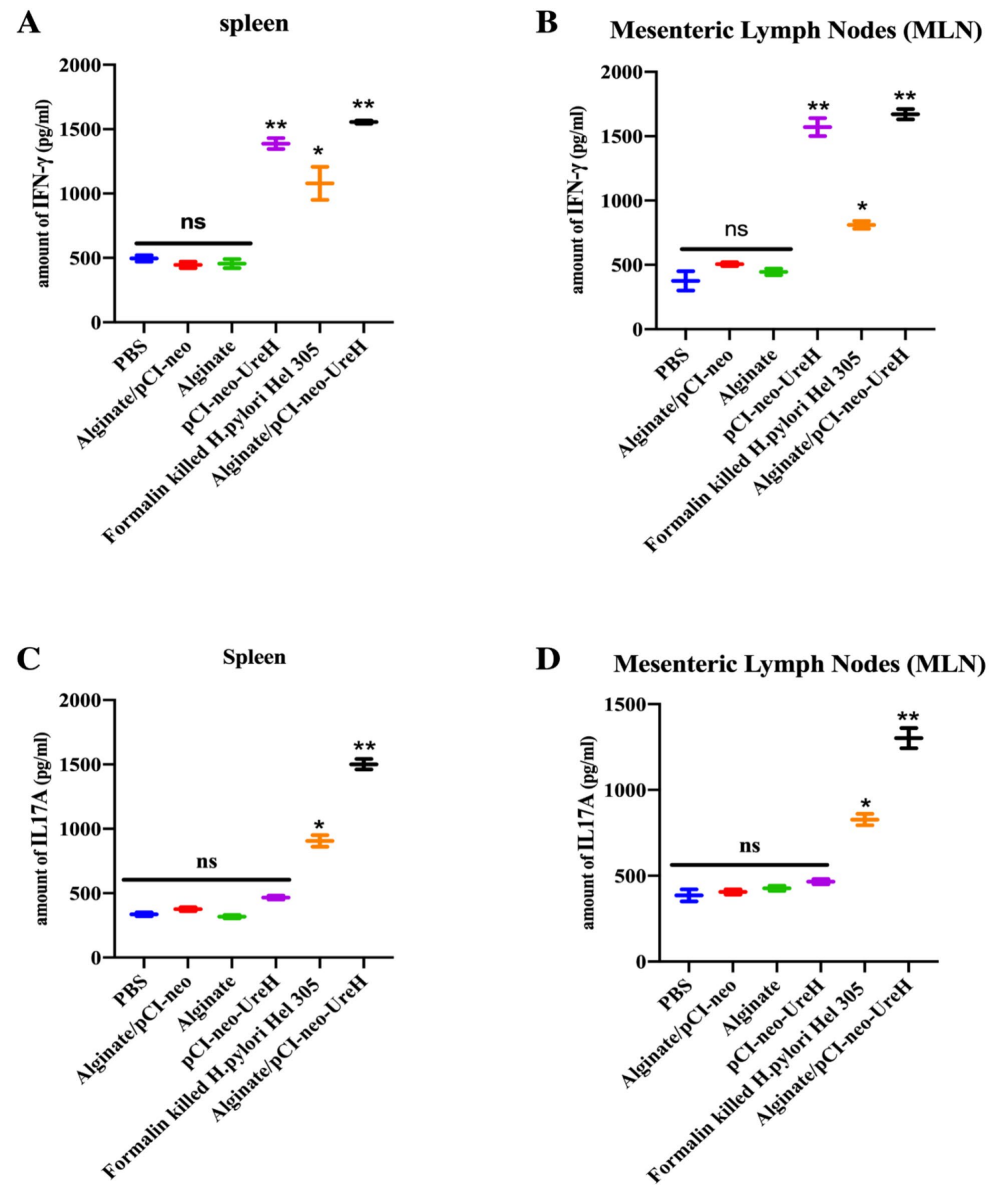
Th1 responses are specific to the *H. pylori* antigen are selectively boosted by oral vaccination with Alginate/pCI-neo-*UreH* and Orally immunized mice with whole-cell killed *H. pylori* Hel 305 and other groups. Two weeks after the previous immunization cycle, cells were extracted from the spleen (**A, C**) and mesenteric lymph nodes (**B, D**). ELISA collected and analysed Supernatants for either IFN-γ (**A, B**) or IL-17 A (**C, D**). Data from three trials with *n* = 5 mice per group and experiment. **p < 0.01, *** < 0.001

Regarding boosting splenocyte Th1 responses, alginate/pCI-neo-*UreH* generated similar reactions to the pCI-neo-*UreH* vaccination (Fig. 3A). Comparing immunization with Alginate alone and formalin-killed *H. pylori* Hel 305 to pCI-neo-*UreH* vaccination, MLNs showed improved specific Th1 responses (Fig. 3B). Though unlike Alginate/pCI-neo-*UreH* (which produced strong Th17 reactions in addition to the IFN-γ responses), the pCI-neo-*UreH* formulation failed to stimulate *H. pylori* antigen-specific Th17 reactions in either spleen (Fig. 3C) or MLNs (Fig. 3D). Alginate/pCI-neo-*UreH* immunization not only improved Th1 responses but also increased antigen-specific spleen and MLN cell proliferation following stimulation. In conclusion, Alginate/pCI-neo-*UreH* immunization enhances antigen-specific Th1 responses in mice infected with *H. pylori*.

### Alginate/pCI-neo-*UreH* treatment orally improves intestinal antigen-specific IgA response to *H. Pylori*

**Fig. 4:**
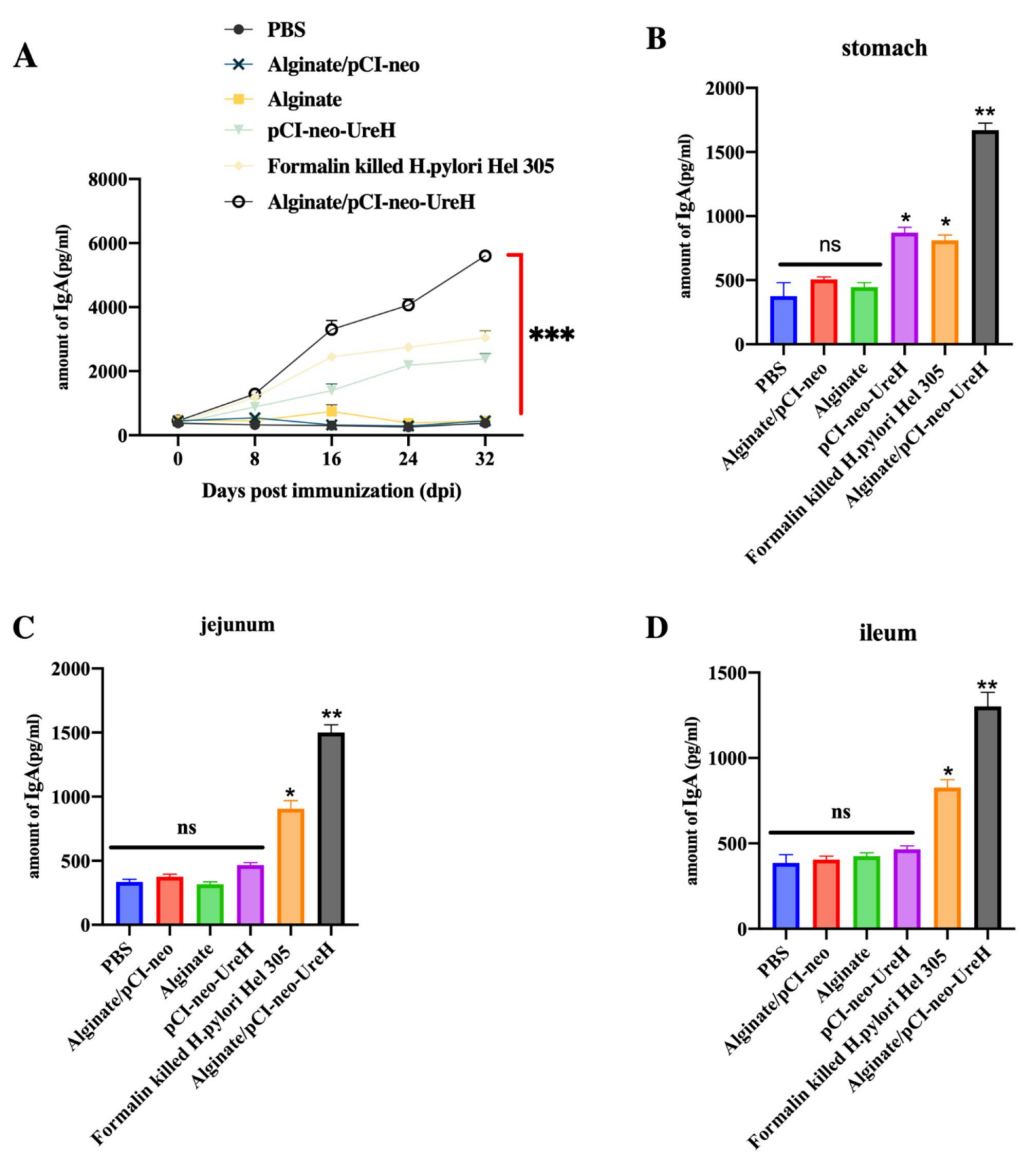
Increased intestine IgA responses to the *H. pylori* antigen by alginate/pCI-neo-*UreH*. Vaccines were Orally administered to mice. ELISA measured specific IgA antibody titers in fecal pellet supernatants before each immunization cycle and two weeks after the final immunization round. **A)** For each group of mice that received the vaccine, the graph line depicts the change in IgA titers over time. Animals were infused to extract the blood from the organs two weeks after the second immunization round, and tissue was recovered. IgA titers specific to *H. pylori* were assessed in extracts from the (**B**) stomach, (**C**) jejunum, and (**D**) ileum. Data from three separate trials with an average of five mice per group and experiment. *p < 0.05, **p < 0.01, ***p < 0.00

After two rounds of oral immunization with various vaccine groups, we next assessed intestinal *H. pylori*-specific IgA responses. After two immunization rounds, oral co-administration of the Alginate/pCI-neo-*UreH* increased antigen-specific IgA reactions in fecal pellet supernatants (Fig. 4A), in contrast to the modest responses seen in mice immunized with the pCI-neo-*UreH* and Alginate alone. Remarkably, after the second immunization round, antigen-specific fecal IgA reactions induced by Alginate/pCI-neo-*UreH* were significantly greater than those produced by the pCI-neo-*UreH* vaccination (Fig. 4A).

Additionally, it was found that vaccination with the Alginate/pCI-neo-*UreH* significantly improved antigen-specific IgA reactions in the stomach (Fig. 4B), the jejunum (Fig. 4C), and the ileum (Fig. 4D) cells compared to the other groups. This was determined by analyzing antigen-specific IgA development from the stomach cells and various segments of the intestine using the Perfext technique. Comparable to the fecal antibody titers, a considerably greater antigen-specific IgA response was detected in ileal extracts after oral vaccination using Alginate/pCI-neo-*UreH*.

**Fig. 5:**
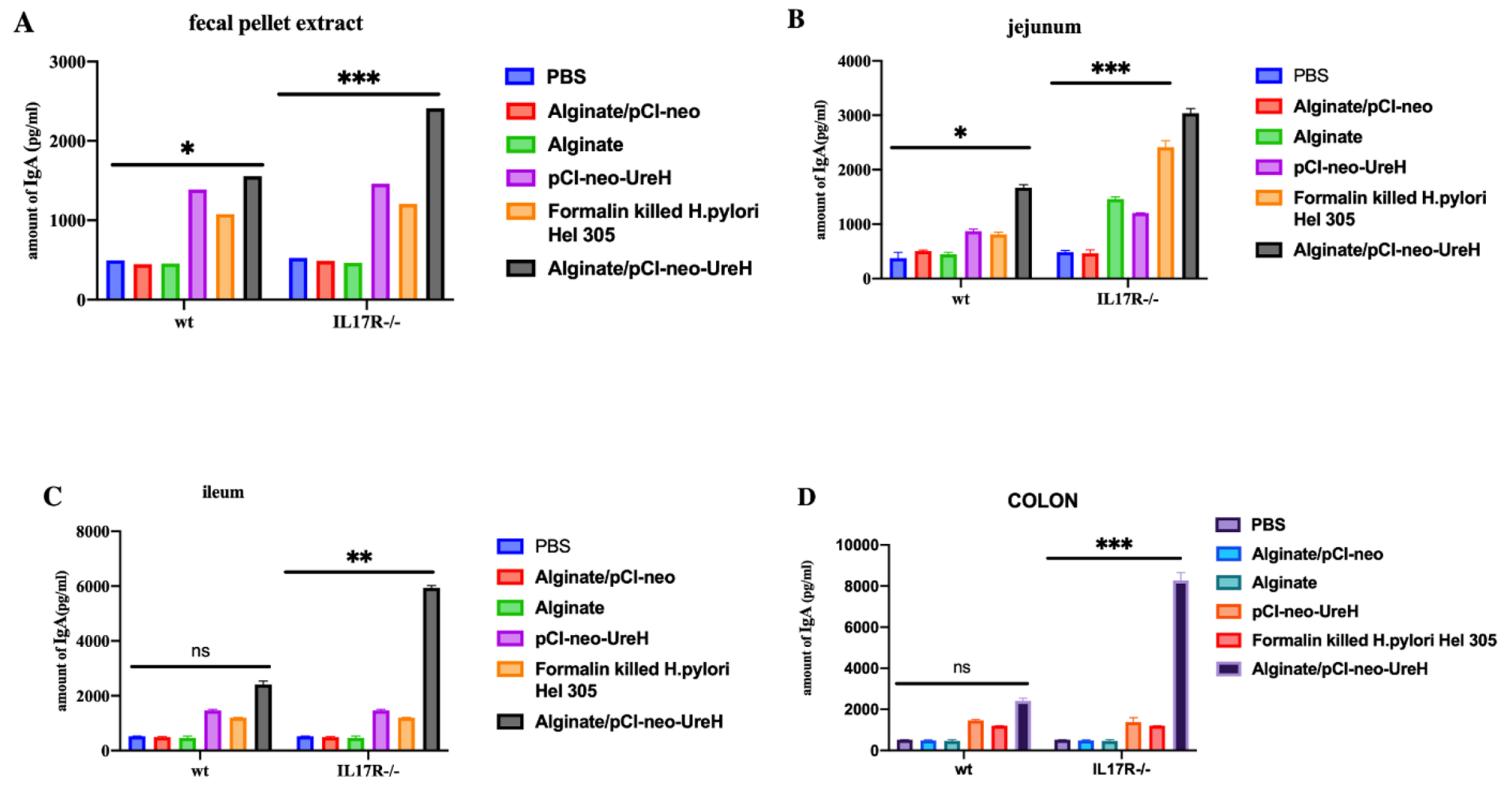
Alginate/pCI-neo-UreH vaccinations were administered intragastrically to WT, IL-17R-/-mice to elicit intestinal, but not systemic, Th1-type responses. Two weeks after the prior vaccination, cells were isolated from the spleen and mesenteric lymph nodes (MLN) and restimulated ex vivo with purified MP305 for 72 h. Supernatants were obtained and subjected to an IgA test from the fecal pellet extract **(A)**, jejunum **(B)**, ileum **(C)**, and colon **(D)**. Results from two distinct investigations, each comprising five animals in each group. *p < 0.05, **p < 0.01; ***p < 0.001

Because Th17 cells are crucial for mucosal antigen-specific IgA responses, it was examined whether the elevated antigen-specific IgA responses resulting from the Alginate/pCI-neo-*UreH* vaccination needed intact IL-17R signalling. Consequently, different vaccination groups were administered to Wild Type and IL-17R-/- mice for immunization. It was shown that Alginate/pCI-neo-*UreH* vaccination could increase antigen-specific IgA reactions in either faecal pellet extracts (Fig. 5A) or jejunal (Fig. 5B), ileal (Fig. 5C), or colonic organ extracts (Fig. 5D). Our results demonstrate that Alginate/pCI-neo-*UreH* vaccination induces antigen-specific IgA reactions without needing IL-17R signalling. The absence of detectable Th17 responses after immunization with the Alginate/pCI-neo-*UreH* vaccine is consistent.

## Discussion

Although significant investment has been allocated to developing an *H. pylori* vaccine in recent years, no such vaccine has yet reached the clinical stage [21, 23, 24]. Since eliciting protective immunity in humans is still challenging, finding safe but successful mucosal vaccines to strengthen local immune defences is a significant obstacle to developing a successful *H. pylori* vaccination [25]. The anti-tumor properties of iNKT cell activator first raised interest in its possible use in medicine [26]. More recently, it has been explored in animal studies for immunization versus HIV [27], influenza [10, 28], and HSV-2 for its propensity to increase local immune responses following mucosal injection [10, 28]. Furthermore, clinical studies have investigated bio-polymer-based vaccinations to stimulate iNKT cells in cases of hepatitis B infections [29] or solid malignancies [36]. Bio-polymer-based vaccination safety has been shown after systemic delivery, indicating they might be a potential mucosal vaccine for individuals [30].

In the current study, intragastric delivery of Alginate/pCI-neo-UreH was shown to be more effective than pCI-neo-UreH and formalin-killed *H. pylori* Hel 305 injection in developing immune defences against *H. pylori* infection. We demonstrated that the protective efficacy of DNA vaccine mucosal target antigen was enhanced by Alginate/pCI-neo-UreH by substantially inducing intestinal and systemic Th1 and Th17 responses. Eliciting antigen-specific IgA responses in ileal tissues and faecal pellet extracts proved even more effective than eliciting these responses in the gut when using alginate/pCI-neo-UreH.

Local Th17 cells were demonstrated by Hirota et al. to be critical for intestinal T-cell-dependent IgA production [31]. Moreover, IL-17 A boosted trans-epithelial IgA synthesis and induced pIgR expression in the intestinal epithelium, according to Cao and colleagues [32]. Also, it was revealed that IL-17 was necessary for establishing cell-mediated vaccination and antigen-specific antibody responses in the serum and mucosa following oral ovalbumin vaccination [10, 33].

Our present data validated the theory that IL17R signaling boosts antigen-specific IgA responses. However, we showed that pCI-neo-UreH and Alginate/pCI-neo-UreH employ two alternative mechanisms to enhance antigen-specific intestinal IgA, suggesting that IL-17 is not essential for IgA transport through the mucosal surface in some instances. Alginate/pCI-neo-UreH also boosted antigen-specific IgA responses in faecal pellets without IL-17R signaling.

In order to identify immunological tests that might replace immunity and to clarify molecular pathways that could be targeted for developing more effective vaccination methods, vaccine regulatory procedures must address how vaccines promote protective immunity [34]. In models of oral infection with *Candida albicans* [36] and lung infection with *Chlamydia muridarum* [35], reduced mucosal Th1 immune responses were seen in IL-17RA-/- mice, indicating the function of the IL-17 receptor in stimulating the Th1 response. Although less clear, IL-1 may contribute to autoimmunity by increasing Th1 responses. Improved Th1 responses may be indirectly brought about by increased IL-1 signaling [37]. To further understand the processes of action of Alginate/pCI-neo-UreH compared to other groups in the stimulation of Th1 responses, we looked at the need for IL17R signaling after vaccination. It was shown that intestinal Th1 responses induced by alginate/pCI-neo-UreH required IL-17R signaling. Remarkably, the need for this signaling pathway was limited to the MLN level and exclusive to Alginate/pCI-neo-UreH since it was not seen in other groups. These results suggest that, in contrast to other groups, Alginate/pCI-neo-UreH produces protective Th1 immune reactions driven by different pathways.

Previous research has shown that following oral delivery of *H. pylori* antigens, *H. pylori* infection is significantly prevented by IFN-γ and IL-17 [38–41]. Here, we prove that IFN-γ was required to stave off *H. pylori* infection brought on by Alginate/pCI-neo-UreH. Finally, we showed that in order to display Alginate/pCI-neo-UreH and initiate protective immune responses, the antigen-presenting molecule CD1d was required. Our results indicate that oral delivery of Alginate/pCI-neo-UreH to iNKT cells occurs via CD1d, which stimulates DCs30 and selectively boosts Th1 responses. The Alginate/pCI-neo-UreH also strongly promotes antigen-specific antibody responses. After vaccination, mice with the Alginate/pCI-neo-UreH *H. pylori* protein may have increased stomach IFN-λ production. This might provide a local pro-inflammatory environment that could activate M1 macrophages and shield animals against *H. pylori* infection.

## Conclusion

Previous research has shown increased inflammatory macrophages and monocytes in the stomach after infection in mice with mucosal immunity [10, 39–41]. Given that intestinal CD4 + T-cell IFN-γ reactions explicitly rely on IL-17R signaling, this is unusual and warrants more explanation. In conclusion, oral Alginate/pCI-neo-UreH immunization induces protective mucosal Th1 immune responses by activating IFN-γ and IL-17R.

### Electronic supplementary material

Below is the link to the electronic supplementary material.

Supplementary Material 1

## Data Availability

The datasets analyzed during the current study are available from the corresponding author upon reasonable request.
