# Supplementary material for: Oral administration of DNA alginate nanovaccine induced immune-protection against Helicobacter pylori in Balb/C mice
Source: BMC Immunol. 2024 Feb 3;25:11. doi: 10.1186/s12865-024-00602-6 (PMC10838413; doi:10.1186/s12865-024-00602-6)

**SUPPLIMENTARY FILE**


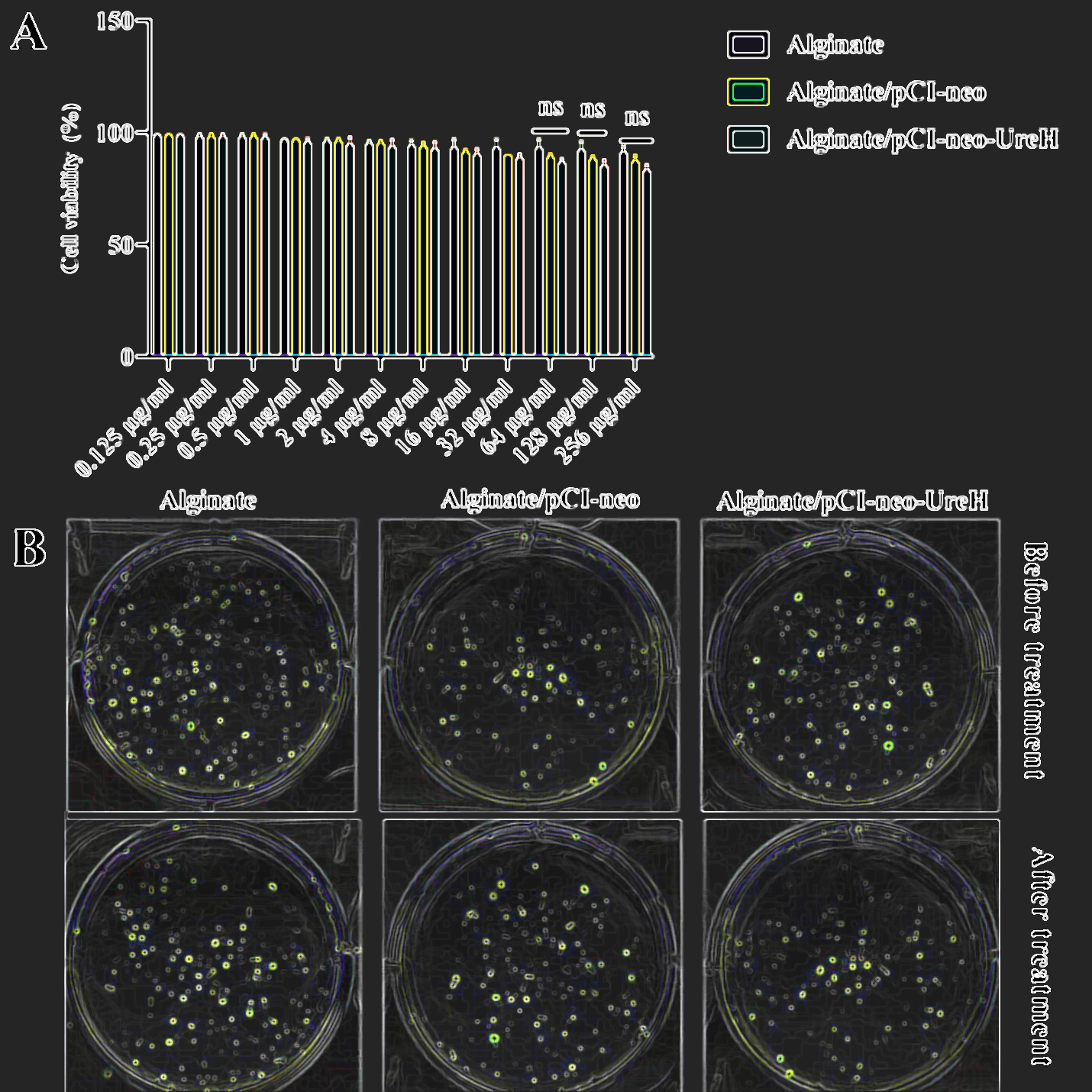


**Figure S1.** Giemsa was used to stain the cell colonies of HEK-293 cells exposed to vaccinations. Three copies of each experiment were run.


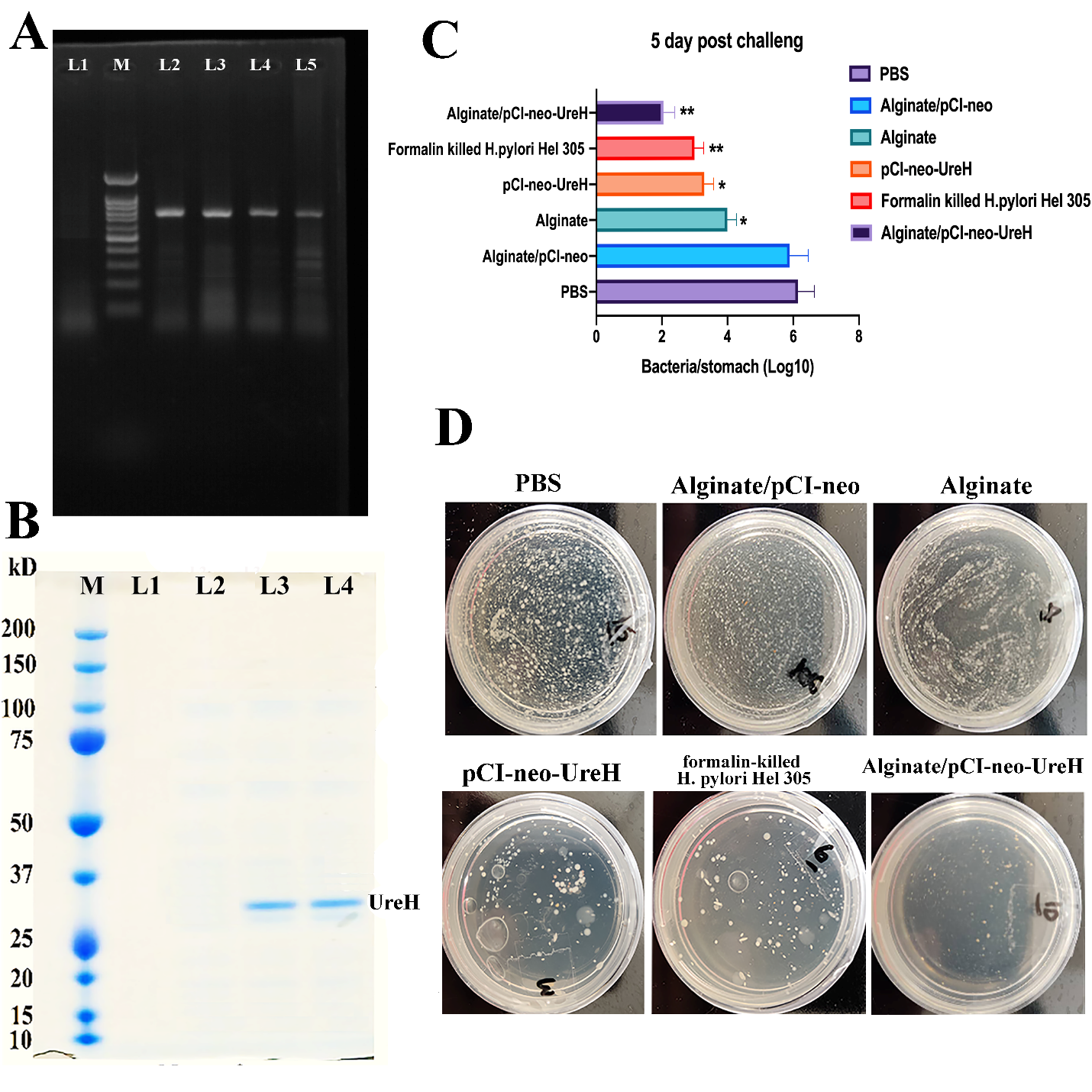


**Figure S2.** Bacterial loads in the blood of mice at 5 days’ post-challenge.

**Full length of gels**


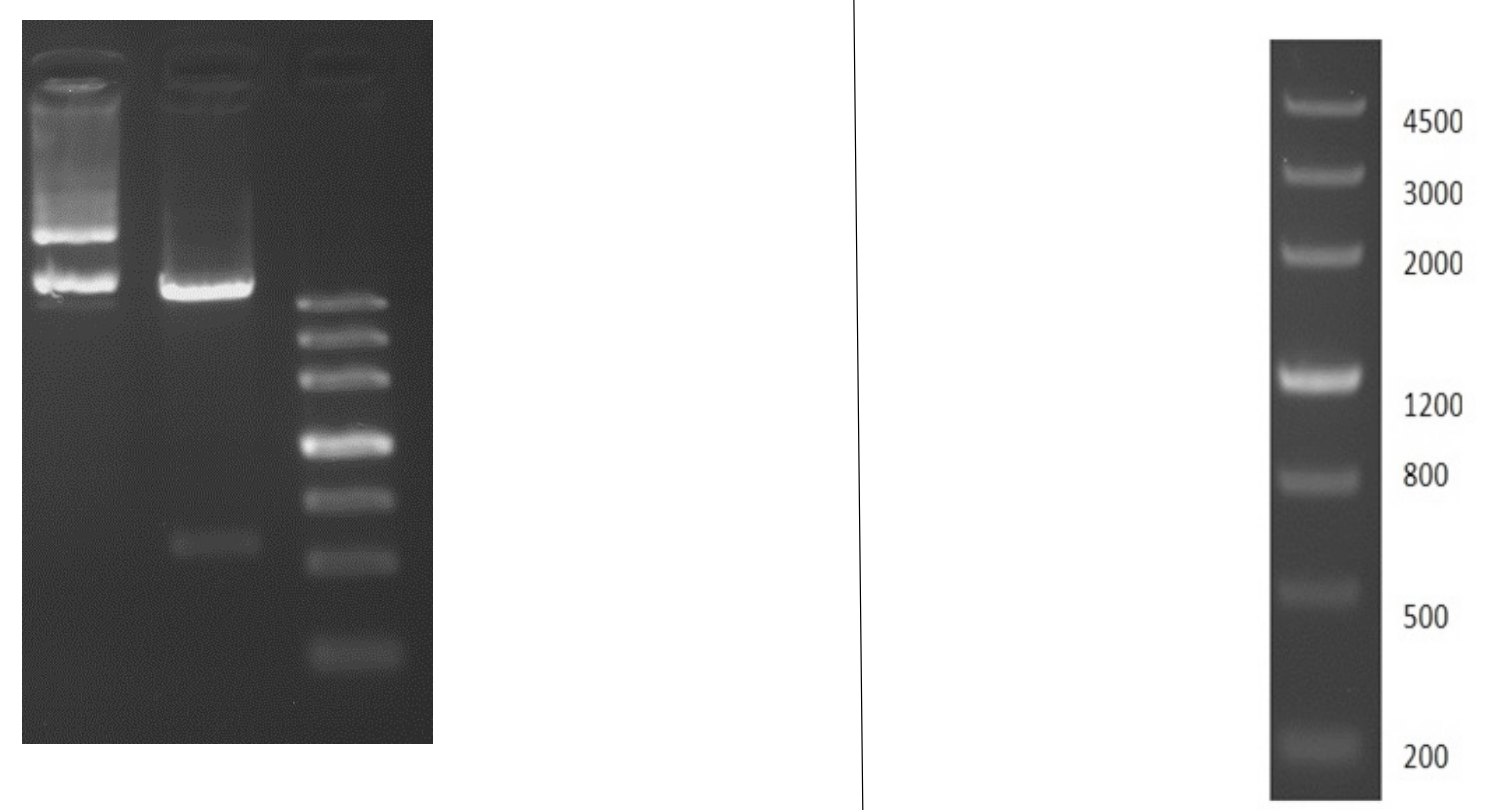


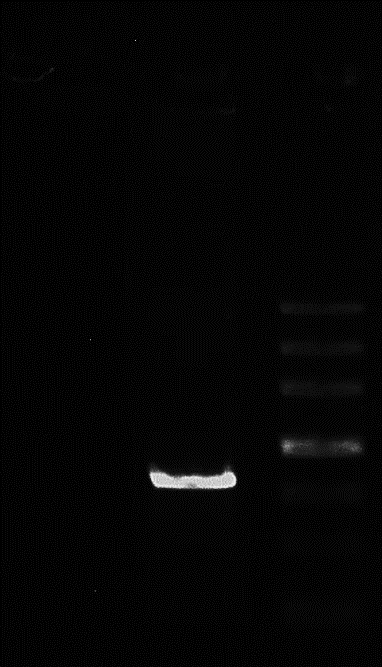


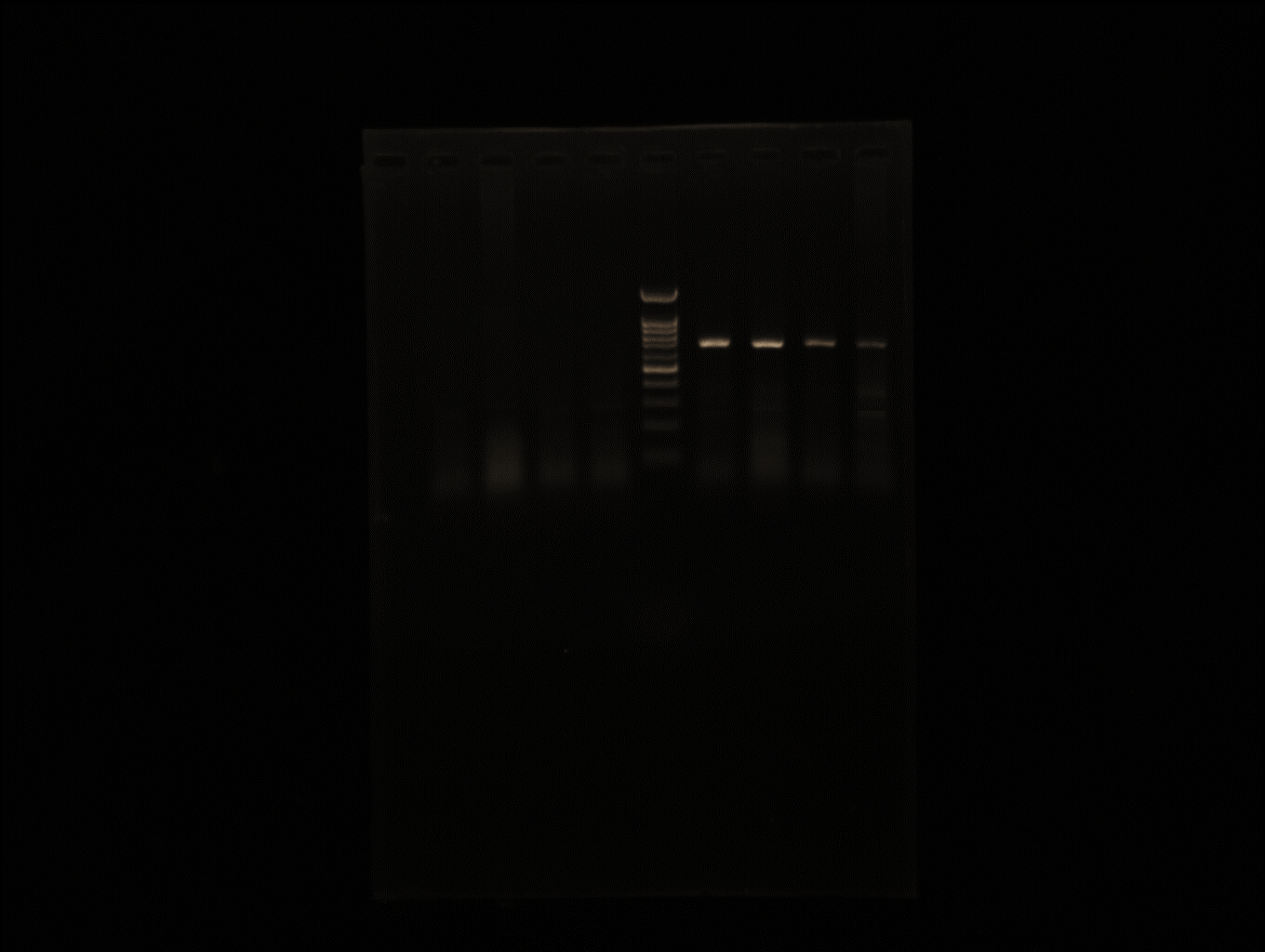


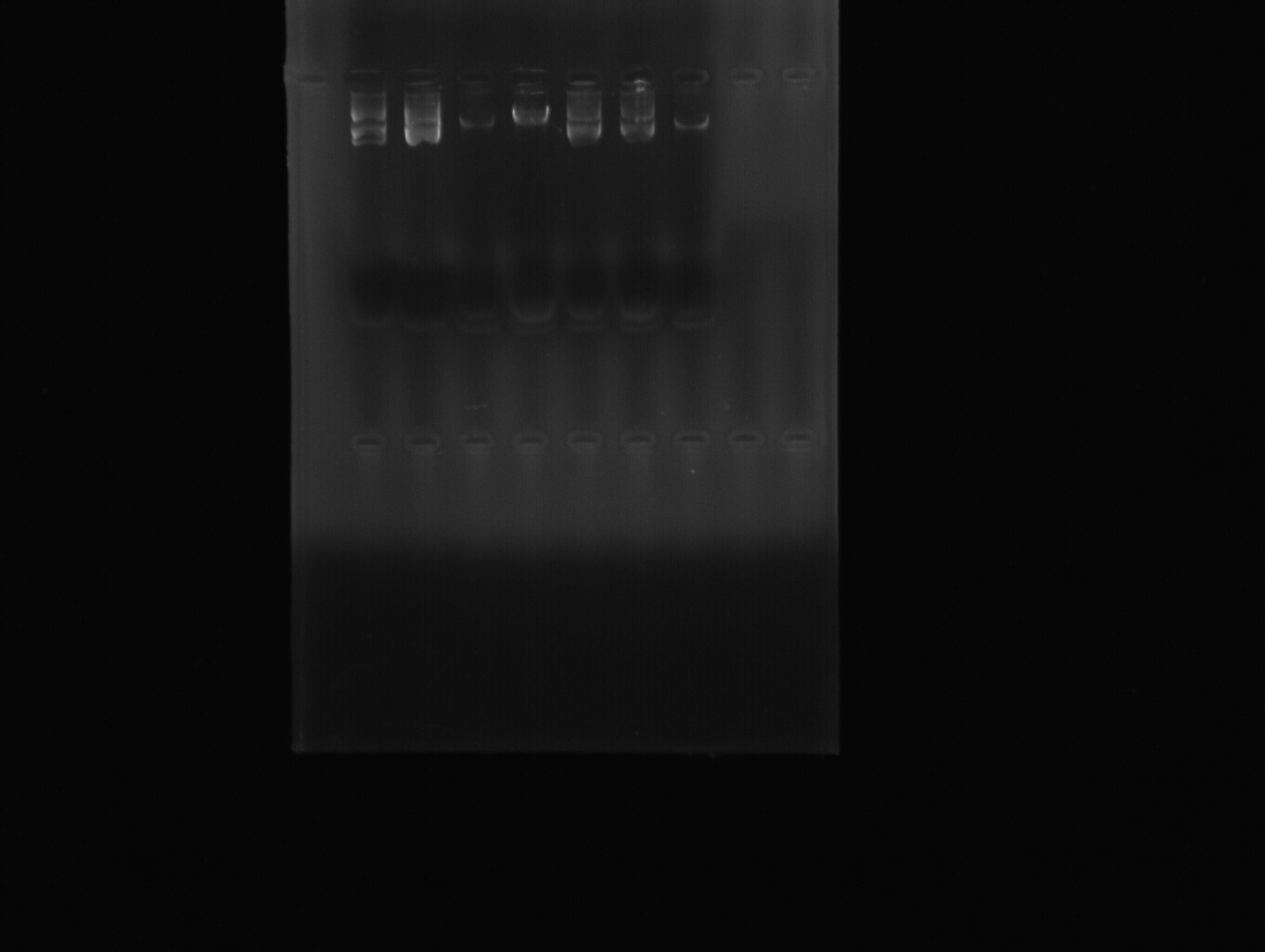


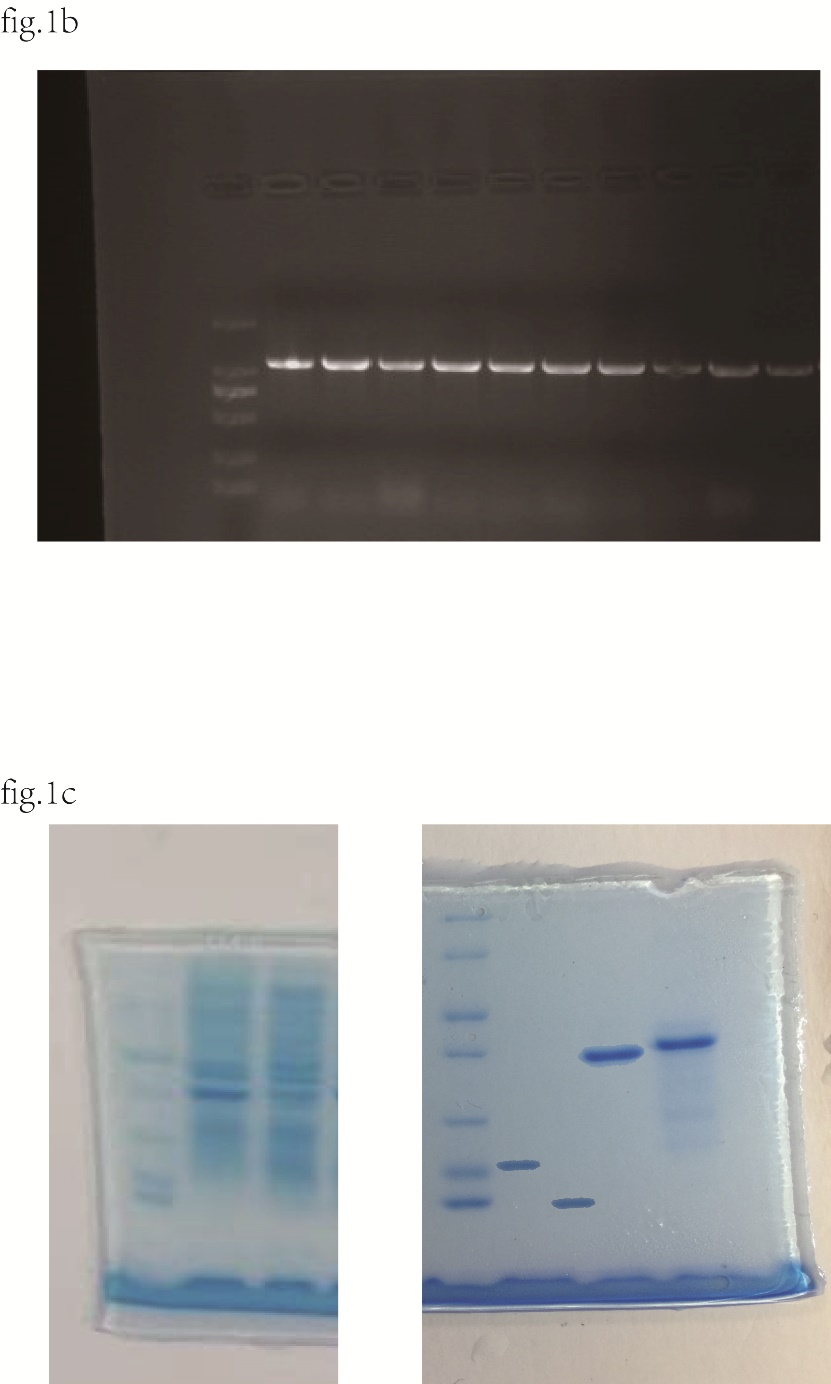

Supplement: Supplementary file 1 — Supplementary Material 1 [file 12865_2024_602_MOESM1_ESM.docx]
